# Pretreatment Neutrophil-to-Lymphocyte Ratio Associated with Tumor Recurrence and Survival in Patients Achieving a Pathological Complete Response Following Neoadjuvant Chemoradiotherapy for Rectal Cancer

**DOI:** 10.3390/cancers13184589

**Published:** 2021-09-13

**Authors:** Chun-Ming Huang, Ming-Yii Huang, Hsiang-Lin Tsai, Ching-Wen Huang, Wei-Chih Su, Tsung-Kun Chang, Yen-Cheng Chen, Ching-Chun Li, Jaw-Yuan Wang

**Affiliations:** 1 Department of Radiation Oncology, Kaohsiung Medical University Hospital, Kaohsiung 80708, Taiwan; 930321@ms.kmuh.org.tw (C.-M.H.); miyihu@kmu.edu.tw (M.-Y.H.); 2 Department of Radiation Oncology, Faculty of Medicine, College of Medicine, Kaohsiung Medical University, Kaohsiung 80708, Taiwan; 3 Department of Radiation Oncology, Kaohsiung Municipal Ta-Tung Hospital, Kaohsiung Medical University, Kaohsiung 80708, Taiwan; 4 Graduate Institute of Medicine, College of Medicine, Kaohsiung Medical University, Kaohsiung 80708, Taiwan; 5 Center for Cancer Research, Kaohsiung Medical University, Kaohsiung 80708, Taiwan; 6 Division of Colorectal Surgery, Department of Surgery, Kaohsiung Medical University Hospital, Kaohsiung Medical University, Kaohsiung 80708, Taiwan; 990367@kmuh.org.tw (H.-L.T.); 1000131@kmuh.org.tw (C.-W.H.); 980278@kmu.edu.tw (W.-C.S.); 990302@kmu.edu.tw (T.-K.C.); 1020421@kmuh.org.tw (Y.-C.C.); 1010363@kmuh.org.tw (C.-C.L.); 7 Department of Surgery, Faculty of Medicine, College of Medicine, Kaohsiung Medical University, Kaohsiung 80708, Taiwan; 8 Graduate Institute of Clinical Medicine, College of Medicine, Kaohsiung Medical University, Kaohsiung 80708, Taiwan; 9 Center for Liquid Biopsy and Cohort Research, Kaohsiung Medical University, Kaohsiung 80708, Taiwan; 10 Master Program for Clinical Pharmacogenomics and Pharmacoproteomics, School of Pharmacy, Taipei Medical University, Taipei 11031, Taiwan; 11 Pingtung Hospital, Ministry of Health and Welfare, Pingtung 90054, Taiwan

**Keywords:** locally advanced rectal cancer, neoadjuvant chemoradiotherapy, neutrophil-to-lymphocyte ratio, prognostic factor, pathological complete response

## Abstract

**Simple Summary:**

Patients with locally advanced rectal cancer who achieve a pathological complete response to neoadjuvant chemoradiotherapy have been associated with excellent long-term prognosis. However, approximately 9% to 12% of patients with a pathological complete response have been reported to experience tumor recurrence and thereby experience poor outcomes. Identifying predictors of recurrence in patients with a pathological complete response is crucial for precise medicine. The neutrophil-to-lymphocyte ratio is a widely available biomarker of systemic inflammation and affects colorectal prognosis. The study aimed to assess the association between neutrophil-to-lymphocyte ratio and oncological outcomes in rectal cancer patients exhibiting a pCR. We found that a pretreatment high neutrophil-to-lymphocyte ratio (≥3.2) was an independent predictor of reduced overall survival and disease-free survival in patients with locally advanced rectal cancer who achieved a pathological complete response to neoadjuvant chemoradiotherapy. Our findings demonstrate that the neutrophil-to-lymphocyte ratio helps identify patients with a pathological complete response who are at high risk of tumor relapse and might facilitate patient selection for precise medicine.

**Abstract:**

The clinical influence of the neutrophil-to-lymphocyte ratio (NLR) in predicting outcomes in patients with locally advanced rectal cancer (LARC) who achieve a pathological complete response (pCR) to neoadjuvant chemoradiotherapy (NACRT) has seldom been investigated. We retrospectively recruited 102 patients with LARC who achieved a pCR to NACRT and the association of NLR status with survival and tumor recurrence in the patients was analyzed. Thirteen patients (12.7%) developed tumor recurrence. A high NLR (≥3.2) was significantly associated with tumor recurrence (*p* = 0.039). The 5-year OS rates in patients with a low NLR and patients with a high NLR were 95.1% and 77.7%, respectively (*p* = 0.014); the 5-year DFS rates in patients with low NLR and patients with a high NLR were 90.6% and 71.3%, respectively (*p* = 0.031). The Cox proportional hazards model indicated that an NLR of ≥3.2 was an independent poor prognostic factor for DFS (hazard ratio [HR] = 3.12, 95% confidence interval [CI] = 1.06–9.46, *p* = 0.048) and OS (HR = 6.96, 95% CI = 1.53–35.51, *p* = 0.013). A pretreatment high NLR (≥3.2) was a promising predictor of reduced OS and DFS in patients with LARC who achieved a pCR to NACRT.

## 1. Introduction

Neoadjuvant chemoradiotherapy (NACRT) followed by radical resection has become the standard treatment in patients with locally advanced rectal cancer (LARC) [1,2]. Patients who achieve a pathological complete response (pCR) to NACRT have been associated with superior long-term prognosis [3,4]. Despite excellent prognosis in patients exhibiting a pCR, approximately 9% to 12% have been reported to experience tumor recurrence [3,5,6]. A pooled analysis of 484 patients with a pCR demonstrated that the 5-year rates of locoregional relapse, distant metastasis, disease-free survival (DFS), and overall survival (OS) were 2.8%, 11.2%, 83.3%, and 87.6%, respectively [7]. According to the literature, systemic relapse has been the leading failure pattern in patients with a pCR [4,5]. Because the benefits of adjuvant chemotherapy in patients with a pCR remain contested, identifying patients with a pCR at high risk of systemic relapse, which might guide the use of adjuvant chemotherapy in such patients following NACRT, is crucial.

Mounting bodies of evidence demonstrate that systemic inflammation influences prognosis and survival in various malignancies [8,9,10], including colorectal cancer (CRC) [11,12]. The neutrophil-to-lymphocyte ratio (NLR) is a widely available biomarker of systemic inflammation and affects CRC prognosis [12,13]. Neutrophils are inflammatory cells that can secrete some cytokines, which can in turn promote angiogenesis, tumor progression, and metastasis [14,15]. Lymphocytes are involved in the immune response to cancer; therefore, lymphopenia is associated with poor prognosis [16]. In other words, the NLR represents the degree of balance between antitumor and protumor effects, which may explain the prognostic effects in numerous malignancies. In focusing on patients with LARC who received NACRT, some studies have suggested that a low NLR is related to increased downstaging and favorable survival rates [17,18]. However, studies reporting the prognostic impacts of NLR status on patients with LARC and a pCR following NACRT are minimal. Accordingly, we conducted this study to assess the association between NLR status and oncological outcomes in such patients exhibiting a pCR.

## 2. Materials and Methods

### 2.1. Ethics Approval Statement

We designed this study in accordance with the Declaration of Helsinki. The institutional review board of our hospital approved the study protocol (KMUHIRB-E(II)-20190280).

### 2.2. Patient Selection

A total of 478 patients with LARC who underwent NACRT between January 2011 and June 2020 were selected from the hospital’s CRC database. Patients who met the following criteria were enrolled: (1) having histologically proven adenocarcinoma; (2) having clinical T3–4 disease, N1–2 disease, or both; (3) having pathologically confirmed ypT0N0 disease; and (4) having tumors located less than 12 cm from the anal verge. Exclusion criteria were as follows: (1) having a history of other malignancy, (2) having loss to follow-up after surgery, (3) having distant metastasis diagnosed initially, and (4) undergoing local tumor excision or palliative resection.

Primary tumors were staged according to magnetic resonance imaging (MRI) of the pelvis and computed tomography (CT) of the abdomen and chest. The patients’ venous blood samples were collected prior to NACRT. The serum carcinoembryonic antigen (CEA) levels, total white blood counts, and neutrophil and lymphocyte counts were collected. The NLR value was derived as the absolute neutrophil count divided by the absolute lymphocyte count. The locations of rectal tumors were dichotomized according to the length between the distal end of the tumor and anal verge: a length of ≤5 cm was considered to indicate low rectal cancer, and a length of 5–12 cm was considered to indicate middle–high rectal cancer [19].

### 2.3. Treatment

All enrolled patients with LARC underwent NACRT, followed by radical resection. The total radiation dose was 45–50.4 Gy, with a daily fraction of 1.8–2.0 Gy. Two concurrent chemotherapeutic regimens were applied in this study: a fluoropyrimidine-only regimen (*n* = 18), which comprised capecitabine; and an oxaliplatin-based regimen (i.e., FOLFOX), which involved oxaliplatin, folinic acid, and 5-fluorouracil (*n* = 84) [20]. 

Approximately 8–12 weeks after the completion of radiotherapy, we performed radical resection with total mesorectal excision (TME). Five patients received abdominoperineal resection, and 97 patients underwent low anterior resection or restorative proctectomy along with coloanal anastomosis. Adjuvant chemotherapy with oral tegafur–uracil (300–400 mg/day) was administered according to recommendations from a shared decision-making platform (https://sdm.patientsafety.mohw.gov.tw/, accessed on 1 June 2016), and the interval of adjuvant chemotherapy was 6 months.

### 2.4. Evaluation and Follow-Up

A pCR was determined when a patient displayed no malignant cells in the primary tumor or lymph nodes. Two independent pathologists assessed tumor responses to NACRT according to a standardized protocol.

The patients were requested to visit a clinic every 3 months during the first 2 years and then once every 6 months thereafter. Evaluations comprised digital rectal examination, chest X-ray, abdominal ultrasound, pelvic MRI or CT scan, colonoscopy, and analysis of serum CEA levels. Positron emission tomography was executed to discriminate tumor relapse from benign lesions where required. We followed up these patients until death or the end of the study period. According to the radiological findings or tissue biopsy, we defined local and distant recurrences as tumor relapse within and outside the pelvis, respectively.

### 2.5. Statistical Analysis

The chi-square test was used to correlate NLR values with clinical parameters. The Kaplan–Meier method was used to create survival curves, and a log-rank test was executed to compare groups. OS was calculated as the period from the date of diagnosis to death from any cause, and DFS was calculated as the period from the date of diagnosis to any recurrence. The Cox regression model was used to evaluate independent prognostic factors related to OS and DFS. Variables with a *p* value of <0.1 in univariate analyses were then assessed in multivariate analyses. The results obtained from the Cox proportional hazard model are reported as hazard ratios (HRs) and 95% confidence intervals (CIs). We conducted receiver operating characteristic (ROC) curve analyses to identify the optimal NLR cutoff value, which should possess the highest sensitivity and specificity in correlation with recurrence. All statistical analyses were executed using JMP software (version 10; SAS Institute, Cary, NC, USA).

## 3. Results

### 3.1. Patient Characteristics

**Figure 1:**
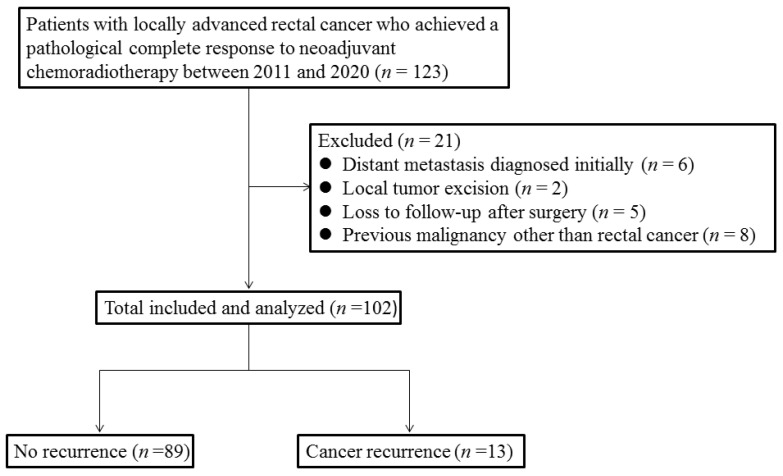
Flow chart of the study population selection.

Of the 123 patients with LARC who exhibited a pCR to NACRT, 21 were excluded (Figure 1). The pCR rate for LARC in our institute was approximately 25.7% (123/478). Among the five patients who achieved a pCR and were lost to follow-up in this study, one patient (20%) was in the high NLR group, and four patients (80%) were in the low NLR group. Among the 102 patients, 65 (63.7%) were men, and 70 (68.6%) had low rectal cancer; the patients had a median age of 63 years (range: 32–87 years). The median dose of radiotherapy was 50 Gy (range: 45–50.4 Gy). A total of 39 (38.2%) patients with a pCR received adjuvant chemotherapy. Table 1 lists the patient characteristics and treatment modalities.

### 3.2. Oncological Outcomes among Patients with pCR

**Figure 2:**
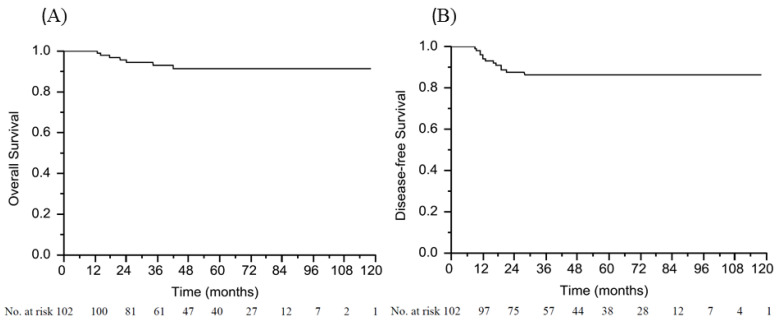
Kaplan–Meier curve depicting overall survival (**A**) and disease-free survival (**B**).

The median follow-up interval was 55 months (range: 11–118 months). In all patients, the 5-year OS was 91.4% and 5-year DFS was 86.3% (Figure 2). Among the 102 patients with pCR, 7 (6.8%) were deceased and 13 (12.7%) experienced disease recurrence. Among the patients with a pCR who developed disease recurrence, 1 simultaneously had local and adrenal relapse and the other 12 patients developed only distant recurrence, which involved 4 liver metastases, 3 lung metastases, 2 retroperitoneal lymph node metastases, 1 brain metastasis, 1 bone metastasis, and 1 peritoneal metastasis.

### 3.3. Optimal NLR Cutoff

**Figure 3:**
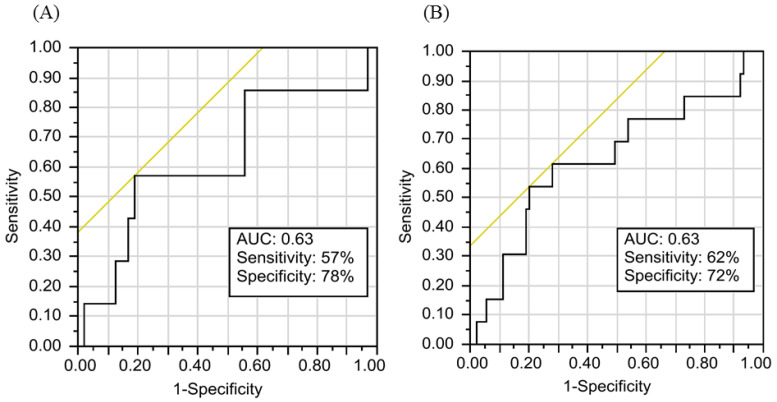
The area under the curve indicating optimal NLR cutoff value. (**A**) AUC for overall survival, (**B**) AUC for disease-free survival.

In the ROC analysis for the NLR cutoff value, the area under the curve (AUC) was 0.63 (*p* = 0.001) for OS, and an NLR of 3.3 exhibited a maximal sensitivity level of 57% and specificity level of 78% (Figure 3A). The AUC was 0.63 (*p* = 0.001) for DFS, and an NLR of 3.2 demonstrated a maximal sensitivity level of 62% and specificity level of 72% (Figure 3B). Accordingly, the optimal NLR cutoff value was 3.3 for OS and 3.2 for DFS. An NLR value of 3.2 was selected for further analysis because prediction of tumor relapse was the primary aim of this study. Finally, an NLR of ≥3.2 was classified as a high NLR level, and an NLR of <3.2 was classified as a low NLR level in this study.

### 3.4. Association between NLR Level and Clinical Variables

The difference in clinical factors according to NLR level is detailed in Table 1. In general, 75 patients (73.5%) were assigned to the low-NLR group, and 27 patients (26.5%) were assigned to the high-NLR group. None of the clinical factors were discovered to be correlated with the NLR, except for tumor relapse status. Our results indicated that the high-NLR group was more likely to develop tumor recurrence (*p* = 0.039).

### 3.5. Association between NLR Level and Survival Outcomes

**Figure 4:**
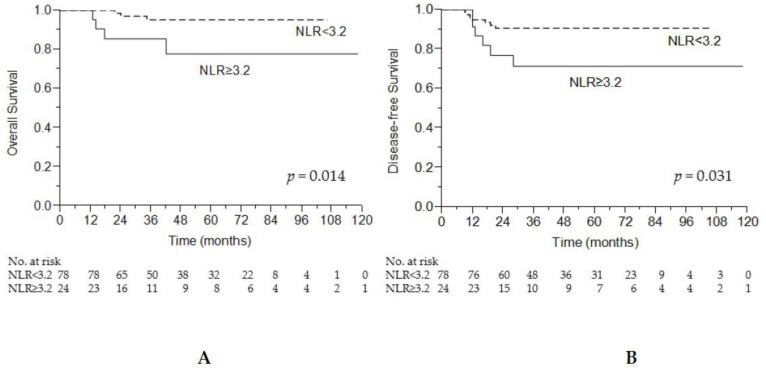
Kaplan–Meier curve depicting overall survival (**A**) and disease-free survival (**B**) according to NLR status.

Our study revealed that patients with a pCR who recorded a high NLR exhibited poorer survival compared with other patients. The 5-year OS rates in the low-NLR (<3.2) and high-NLR (≥3.2) groups were 95.1% and 77.7%, respectively (*p* = 0.014; Figure 4A). The 5-year DFS rates in the low-NLR (<3.2) and high-NLR (≥3.2) groups were 90.6% and 71.3%, respectively (*p* = 0.031; Figure 4B).

Table 2 presents the results of the univariate and multivariate analyses of OS. In the univariate analysis, a high pretreatment NLR was a significant predictor of decreased OS (HR = 5.34, 95% CI = 1.17–27.12, *p* = 0.031). Low rectal cancer (*p* = 0.064) and a preoperative fluoropyrimidine-only regimen (*p* = 0.076) were correlated with decreased OS. In the multivariate analysis, the high-NLR group demonstrated significantly lower OS (HR = 6.96, 95% CI = 1.53–35.51, *p* = 0.013). The results of the univariate and multivariate analyses of DFS are presented in Table 3. In the univariate analysis, the high-NLR group was significantly associated with decreased DFS (HR = 3.13, 95% CI = 1.01–9.44, *p* = 0.041), and clinically positive nodal disease (*p* = 0.066), and a preoperative fluoropyrimidine-only regimen (*p* = 0.071) were correlated with reduced DFS. In the multivariate Cox proportional hazards model, a high NLR (≥3.2) was an independent poor prognostic factor for DFS (HR = 3.12, 95% CI = 1.06–9.46, *p* = 0.048). To evaluate the loss of information caused by dichotomization of continuous predictor variables, we used NLR as a continuous variable to conduct multivariate analyses. We found that elevated NLR values were associated with poor OS (HR = 1.24, 95% CI = 1.01–1.46, *p* = 0.038). Similarly, elevated NLRs resulted in unfavorable DFS (HR = 1.23, 95% CI = 1.06–1.41, *p* = 0.012).

## 4. Discussion

Our study indicated that a high NLR (≥3.2) was associated with decreased OS and DFS in patients with a pCR to NACRT and TME. According to our review of the literature, this is the first study to clarify the prognostic effects of the NLR on patients with LARC who achieved a pCR to NACRT. Most studies have emphasized the identification of pCR following NACRT and have reported that a pCR is correlated with excellent prognosis [6,7,21,22]. However, approximately 10% of patients with a pCR have been reported to develop tumor relapse and thereby experience poor outcomes [5,7,23]. Identifying predictors of recurrence in patients with a pCR is crucial for precise medicine. Therefore, our findings demonstrate that the NLR status helps identify patients with a pCR who are at high risk of tumor relapse and might facilitate patient selection for adjuvant chemotherapy.

Consistent with previous reports, our results reveal that patients with LARC who achieved a pCR to NACRT exhibited favorable oncological outcomes. The 5-year OS and DFS rates were 91.4% and 86.3%, respectively. A meta-analysis revealed that in the pCR group, the 5-year OS and DFS rates were 92.9% and 86.9%, respectively [4]. Sun et al. analyzed 118 patients with a pCR and reported that the 5-year OS and DFS rates were 94.7% and 88.1%, respectively [23]. Although patients who achieved a pCR following NACRT obtain a favorable prognosis, a small subsection of patients with a pCR ultimately experience tumor relapse. Our findings suggest that the patterns of treatment failure in patients who achieved a pCR after NACRT and TME were mainly distant metastases. A pooled analysis of 484 patients with a pCR revealed that the 5-year rates of distant metastasis and local recurrence were 11.2% and 2.8%, respectively [7]. Capirci et al. recruited 566 patients with a pCR from 61 centers and reported that the distant metastasis and locoregional recurrence rates were 8.9% and 1.6%, respectively [3]. In our study, the distant metastasis and local recurrence rates were 12.7% and 1%, respectively, which are comparable to previously published reports. A predominance of distant recurrence in patients who undergo a pCR after NACRT may imply that intensified systemic therapy is necessary.

The benefits of adjuvant chemotherapy in patients with LARC and who experienced a pCR to NACRT remain controversial. Some studies have indicated that patients with a pCR benefited from adjuvant chemotherapy, exhibiting an increase in OS in comparison with such patients without adjuvant chemotherapy [24,25]. Some researchers have advocated omitting adjuvant chemotherapy in patients with a pCR because of their excellent survival, but others have supported the administration of adjuvant chemotherapy to reduce the risk of disease recurrence [26]. A reliable biomarker for risk stratification may resolve this dispute. Our findings suggest that an increased NLR was associated with tumor relapse and a lower survival rate in patients with LARC who exhibited a pCR following NACRT. On the basis of the NLR-based risk stratification, the administration of adjuvant chemotherapy to patients with a pCR despite a high risk of tumor relapse (i.e., high NLR) can not only maximize the treatment efficacy but also avoid overtreatment in patients at low risk of recurrence (i.e., low NLR). Therefore, the application of the NLR in the prediction of post-pCR tumor recurrence warrants further prospective randomized studies.

In our study, adjuvant chemotherapy did not improve OS and DFS in patients with a pCR after NACRT. As mentioned above, routine administration of adjuvant chemotherapy did not benefit patients with a pCR. Therefore, some researchers have suggested that total neoadjuvant therapy (TNT), which combines chemotherapy with NACRT prior to surgery, can enhance tumor regression and treatment compliance, and improve DFS in patients with LARC. Cercek et al. demonstrated that rates of chemotherapy compliance and complete response were significantly higher in the TNT group compared to those in the NACRT and adjuvant chemotherapy group [27]. A meta-analysis suggested that TNT was associated with superior rates of pCR and DFS compared with NACRT followed by radical resection and adjuvant chemotherapy [28]. TNT has been considered to be effective in the reduction of distant metastases because of the early administration of potent chemotherapy [29,30]. In this study, distant metastases were the majority of treatment failure, and thus TNT may improve oncological outcomes by decreasing distant metastases. Although studies have supported that TNT resulted in superior outcomes, overtreatment of patients with low-risk disease should be avoided. The NLR is a potential biomarker in the identification of patients at high risk of recurrence, and thus can optimize the use of TNT.

The reason why a high NLR correlates with poor oncological outcomes remains unclear. One possible underlying mechanism may involve tumor-infiltrating immune cells that mainly consist of neutrophils and lymphocytes. Tumor-associated neutrophils are mediated by certain cytokines released by cancer cells, and such neutrophils have been associated with local inflammation, angiogenesis, and cancer migration, which suppress antitumor immunity and thereby promote tumor progression and metastasis [31,32]. Diefenhardt et al. analyzed 1,236 patients with rectal cancer who received NACRT and discovered that peripheral neutrophilia was correlated with reduced OS and DFS [33]. By contrast, tumor-infiltrating lymphocytes (TILs) are involved in the antitumor immune response, which is primarily mediated by T-cell-dependent cellular immunity [34]. The density of TILs has been proven to be correlated with favorable survival and tumor response to NACRT in patients with rectal cancer [35,36]. Additionally, studies have indicated that lymphopenia was associated with a low density of TILs, and a low absolute lymphocyte count predicted poor pathological response and low survival in patients with LARC who received NACRT [37]. Therefore, the NLR indicates the degree of balance between antitumor and protumor immunity, which may explain why the ratio correlates well with prognosis in rectal cancer. In the current study, a high NLR (≥3.2) was significantly associated with an elevated risk of distant recurrence and lower survival in patients with LARC who had a pCR following NACRT. Our findings are consistent with those of studies suggesting that the NLR is correlated with oncological outcomes in patients with a pCR. In fact, several studies and meta-analyses have supported the prognostic and predictive values of NLR in patients with rectal cancer with or without NACRT. A meta-analysis by Hamid et al. demonstrated that high NLR was a poor prognostic factor and associated with unfavorable OS (HR = 1.92, 95% CI = 1.60–2.30, *p* < 0.001) and DFS (HR = 1.83, 95% CI = 1.51–2.22, *p* < 0.001) [38]. Besides, the meta-analysis showed that low NLR was a significant predictor of pCR (OR = 1.62, 95% CI = 1.16–2.27, *p* = 0.004). In this study, we demonstrated that NLR was a prognostic indicator of OS and DFS in patients with LARC who had a pCR following NACRT. The prognostic value of NLR in the patients with LARC who have achieved a pCR to NACRT is seldom reported.

One controversial question pertains to the optimal NLR cutoff. A study in which 199 patients with LARC were dichotomized according to NLR status revealed that the 5-year OS was significantly lower in patients with a high NLR (≥2.8) than it was in those with a low (<2.8) NLR (43.7% vs. 71.7%, *p* = 0.002) [17]. In another study analyzing 115 patients with LARC who received NACRT, the median survival rate was significantly lower in the high-NLR (>5) group than it was in the low-NLR (≤5) group (18.8 vs. 54.4 months, *p* < 0.001) [39]. However, a meta-analysis of 7,553 patients (with NLR cutoff levels ranging from 1.75 to 5) with rectal cancer revealed that an NLR of ≥4 was not associated with OS or DFS [38]. Overall, most studies have focused on the impacts of the NLR on downstaging or survival in patients with LARC following NACRT, and researchers have usually demonstrated correlations between high NLR values and poor oncological outcomes [17,18,40]. However, the correlation between the NLR and the prognostic outcomes in patients with LARC who achieve a pCR following NACRT is seldom reported, and identification of an optimal NLR cutoff even less so. We conducted ROC analyses for determining the optimal NLR cutoff to identify the patients with a pCR at high risk of tumor relapse; the AUC values for OS and DFS were both 0.63, which is consistent with previous study findings [17,18]. In the current study, the NLR cutoff for DFS with highest sensitivity (62%) and specificity (72%) was 3.2. Additional multi-institutional studies are necessary not only to identify an optimal cutoff for the NLR but also to validate our findings. ROC curves were often used to select optimal cutoff point for NLR. However, it is well known that dichotomization of continuous predictor variables may lead to loss of information and make impossible to compare similar studies with different cutoffs. Therefore, while its dichotomization is justified by the resulting simplification of both results’ interpretation and clinical decision-making process, it would be useful to use NLR also as a continuous variable in multivariate analyses. In this study, we used NLR as a continuous variable to conduct multivariate analyses. We found that elevated NLR values were associated with poor OS (HR = 1.24, 95% CI = 1.01–1.46, *p* = 0.038) and poor DFS (HR = 1.23, 95% CI = 1.06–1.41, *p* = 0.012).

We acknowledge several limitations to this study. First, this was a retrospective study at a single institute and is thus subject to potential selection bias and a relatively small sample size. Second, the NLR is a surrogate marker of systemic inflammation and may be influenced by certain circumstances, such as nutritional status, inflammatory diseases, metabolic diseases, and the administration of anti-inflammatory medicine [41,42]. Despite these limitations, we believe that our findings can help guide patient stratification in the pCR group following NACRT and radical resection.

## 5. Conclusions

Our study revealed a pretreatment high NLR (≥3.2) to be an independent prognostic factor of poor OS and DFS in patients with LARC who have achieved a pCR to NACRT. On the basis of our NLR stratification, patients with a pCR and a high pretreatment NLR might be suggested for administration of TNT or adjuvant therapy due to their relatively high distant relapse rate. Additional investigations are warranted to verify the association of prognostic outcomes with NLR status in patients with a pCR and to clarify the role of NLR status in guiding the selection for TNT or adjuvant therapy in patients with a pCR.

## Figures and Tables

**Table 1:** Patient characteristics of pathological complete response patients (*n* = 102).

Variables	All Patients (%)	Low NLR (%)	High NLR (%)	*p*-Value
Sex				0.303
Male	65 (63.7)	50 (63.7)	15 (55.6)	
Female	37 (36.3)	25 (36.3)	12 (44.4)	
Age, years				0.153
≤60	34 (33.3)	22 (33.3)	12 (44.4)	
>60	68 (66.7)	53 (70.7)	15 (55.6)	
Tumor location				0.797
Low	70 (68.6)	52 (69.3)	18 (66.7)	
Middle–high	32 (31.4)	23 (30.7)	9 (33.3)	
Histopathology				0.291
Adenocarcinoma	99 (97.1)	72 (96)	27 (100)	
Mucinous adenocarcinoma	3 (2.9)	3 (4)	0 (0)	
cT stage				0.647
3	91 (89.2)	67 (89.3)	24 (88.9)	
4	11 (19.8)	8 (10.7)	3 (11.1)	
cN				0.777
Negative	28 (27.5)	22 (29.3)	6 (22.2)	
Positive	74 (72.5)	53 (70.7)	21 (77.8)	
Pretreatment CEA, ng/mL				0.225
<5	74 (72.5)	52 (69.3)	22 (81.5)	
≥5	28 (27.5)	23 (30.7)	5 (18.5)	
Preoperative chemotherapy regimen				0.451
Oxaliplatin-based	84 (82.4)	60 (80)	24 (88.9)	
Fluoropyrimidine only	18 (17.6)	15 (20)	3 (11.1)	
Type of surgery				0.903
LAR	97 (95.1)	71 (94.7)	26 (96.3)	
APR	5 (4.9)	4 (5.3)	1 (3.7)	
Adjuvant chemotherapy				0.675
Yes	39 (38.2)	29 (38.7)	10 (37)	
No	63 (61.8)	46 (61.3)	17 (63)	
All Recurrence				0.039
Yes	13 (12.7)	7 (9)	6 (25)	
No	89 (87.3)	71 (91)	18 (75)	
Local recurrence				0.07
Yes *	1 (1)	0 (0)	1 (95.8)	
No	0 (99))	78 (100)	23 (4.2)	
Distant recurrence				0.039
Yes	13 (12.7)	7 (9)	6 (25)	
No	89 (87.3)	71 (91)	18 (75)	

NLR: neutrophil-to-lymphocyte ratio; LAR: low anterior resection; APR: abdominoperineal resection. * One patient experienced local and distant recurrence simultaneously.

**Table 2:** Univariate and multivariate analysis for overall survival.

Variables	Univariate			Multivariate		
	HR	95% CI	*p* Value	HR	95% CI	*p* Value
Sex			0.770			
Male	1					
Female	1.25	0.24–5.69				
Age			0.696			
≤60	1					
>60	0.74	0.16–3.75				
Tumor location			0.064			0.104
Low	1			1		
Middle–high	0.39	0.32–6.12		0.84	0.37–6.52	
Histopathology			0.488			
Adenocarcinoma	1					
Mucinous adenocarcinoma	3.92	0.45–6.73				
cT stage			0.524			
3	1					
4	2.12	0.11–12.61				
cN			0.915			
Negative	1					
Positive	0.91	0.19–6.38				
Pretreatment CEA, ng/mL			0.371			
<5	1					
≥5	2.01	0.39–9.14				
Preoperative chemotherapy regimen			0.076			0.146
Oxaliplatin-based	1			1		
Fluoropyrimidine only	3.83	0.62–8.13		1.08	0.93–8.27	
Type of surgery			0.439			
LAR	1					
APR	2.54	0.13–14.95				
Adjuvant chemotherapy			0.359			
Yes	1					
No	0.48	0.11–2.49				
Pretreatment NLR			0.031			0.013
<3.2	1			1		
≥3.2	5.34	1.17–27.12		6.96	1.53–35.51	

NLR: neutrophil-to-lymphocyte ratio; LAR: low anterior resection; APR: abdominoperineal resection; HR: hazard ratio; CI: confidence interval.

**Table 3:** Univariate and multivariate analysis for disease-free survival.

Variables	Univariate			Multivariate		
	HR	95% CI	*p* Value	HR	95% CI	*p* Value
Sex			0.902			
Male	1					
Female	1.07	0.32–3.21				
Age			0.148			
≤60	1					
>60	0.44	0.14–1.34				
Tumor location			0.187			
Low	1					
Middle–high	0.45	0.06–1.48				
Histopathology			0.401			
Adenocarcinoma	1					
Mucinous adenocarcinoma	2.72	0.14–13.82				
cT stage			0.466			
3	1					
4	1.83	0.28–6.86				
cN			0.066			0.114
Negative	1			1		
Positive	4.65	0.91–84.75		3.89	0.76–71.14	
Pretreatment CEA, ng/mL			0.751			
<5	1					
≥5	1.21	0.32–3.72				
Preoperative chemotherapy regimen			0.071			0.182
Oxaliplatin-based	1			1		
Fluoropyrimidine only	2.31	0.22–7.93		1.37	0.32–6.76	
Type of surgery			0.653			
LAR	1					
APR	1.65	0.09–8.39				
Adjuvant chemotherapy			0.117			
Yes	1					
No	0.41	0.13–1.25				
Pretreatment NLR			0.041			0.048
<3.2	1			1		
≥3.2	3.13	1.01–9.44		3.12	1.06–9.46	

NLR: neutrophil-to-lymphocyte ratio; LAR: low anterior resection; APR: abdominoperineal resection; HR: hazard ratio; CI: confidence interval.

## Data Availability

The data used to support the findings of this study are included within the article and the data sources are available from the corresponding author upon request.
